# NO DIFFERENCE IN FINAL RADIOGRAPHIC FRACTURE DISPLACEMENT IN TIBIAL PLATEAU FRACTURES: IMN VS ORIF

**DOI:** 10.51894/001c.123056

**Published:** 2024-08-30

**Authors:** Adrian Olson

**Affiliations:** 1 Orthopedic Surgery Ascension Macomb Oakland

## 69

### INTRODUCTION

Currently the prevailing surgical treatment for displaced tibial plateau fractures in adults is approached through open reduction and internal fixation (ORIF) using a combination of plates and screws. Obtaining adequate surgical fracture reduction is essential, and historically there has been hesitation in the use of intramedullary nail (IMN) constructs in the treatment of displaced tibial plateau fractures due to a concern over its ability to maintain joint reduction in such cases.

### OBJECTIVES

Retrospective cohort study investigating displaced tibial plateau fractures treated by IMN versus ORIF for comparative outcome analysis of radiographic postoperative fracture reduction and patient clinical outcomes based on type of fixation performed.

### METHODS

From July 2012 to July 2022, 58 patients with AO 41 C2 and C3 type tibial plateau fractures were treated at a level-one trauma center. Two matched groups were included (28 suprapatellar IMN; 30 ORIF). Preoperative fracture displacement was measured on standard radiographs and CT scan to determine extent of condylar widening and joint depression. Final radiographic healing and displacement was measured at 2, 6, and 12 months follow-up. Clinical outcomes during these time intervals included time to range of motion >90° and time to full weight bearing.

### RESULTS

The two groups demonstrated no demographic differences in age, BMI, treatment with external fixator, decompressive fasciotomy, fracture classification, or initial fracture displacement. The preoperative joint line depression in the IMN vs ORIF group was 5.6 mm and 8.2 mm, respectively (p=0.054). Preoperative condylar widening was 5.8mm (IMN) vs 7.2mm (ORIF) (p=0.461). There were no significant differences in final healed joint line depression or condylar widening between the groups at 2, 6, and 12 months postoperatively. The absolute joint line subsidence and condylar widening was on average <1 mm in both groups at 12 months.

### CONCLUSION

There was no statistically significant difference in final healed joint line displacement or condylar widening in tibial plateau fractures treated with suprapatellar IMN compared to a matched ORIF group. This technique may be useful in circumstances where ORIF of the tibial plateau fracture places the soft tissue envelope at unacceptable risk or where an intramedullary implant is otherwise preferred.

